# Supplementary material for: Community-developed checklists for publishing images and image analyses
Source: ArXiv. 2023 Sep 14:arXiv:2302.07005v2. Originally published 2023 Feb 14. Preprint. [Version 2] (PMC9949169)
Supplement: 1 [file NIHPP2302.07005V2-supplement-1.pdf]

Supplemental materials

Supplemental figure 1. *Alternative layout Checklist for image Publishing*

Checklist for image publishing

Image format

Item	Level
Focus on relevant image content (e.g. crop, rotate, resize)	Minimal
Separate individual images	
Show example image used for quantifications	
Indicate position of zoom-view/inset in full-view/ original image	
Show images of the range of described phenotype	

Image colors and channels

Item	Level
Annotation of channels (staining, marker etc.) visible	Minimal
Adjust brightness/contrast, report adjustments, use uniform color-scales	
Image comparison: use same adjustments	
Multi-color images: accessible to color blind	
Channel color high visibility on background	
Provide grey-scale for each color channel	Recommended
Provide color scales for intensity values (greyscale, color, pseudo color...)	
Pseudo-colored images: additionally provide greyscale version for comparison.	Ideal
Gamma adjustments: additionally provide linear-adjusted image for comparison	

Image annotation

Item	Level
Add scale information (scale bar, image length)	Minimal
Explain all annotations	
Legible annotations (point size, color)	
Annotations should not obscure key data	
Annotate image dimensions (z-distance in image stacks), image pixel size, imaging intervals (time-lapse in movies)	Recommended
Annotate imaging details e.g., exposure time	Ideal

Image availability

Item	Level
Images are shared (lossless compression/microscope images)	Minimal
Image files are freely downloadable (public database)	Recommended
Image files are in dedicated image database (added value database or image archive)	Ideal

Supplemental figure 2. *Alternative layout Checklist for image Analysis Publishing*

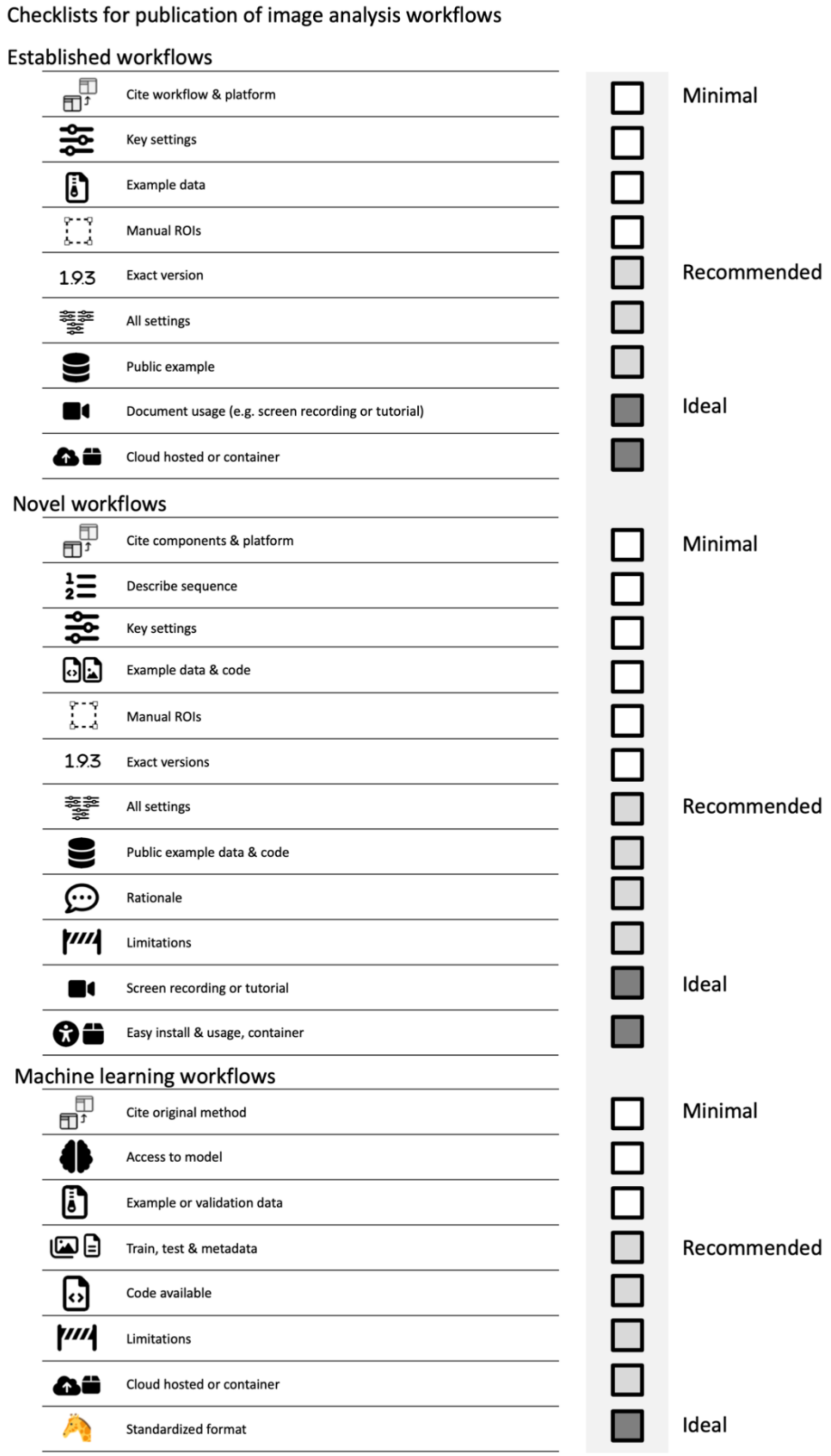

Supplemental figure 3. *Overview of current repositories that accept image data. (Cimini, 2023)*

Repository	Zenodo	Figshare / Figshare+	Dryad	Bioimage Archive	Image Data Repository	Broad Bioimage Benchmark Collection	The Cell Painting Gallery
URL	https://zenodo.org/	https://figshare.com/	https://datadryad.org/	https://www.ebi.ac.uk/bioimage-archive/	https://idr.openmicroscopy.org/	https://broad.io/BBBC	https://broad.io/cellpaintinggallery
Qualifications	"All the digital artefacts"	Research data outputs	Non-human identifiable data of any kind that authors are willing to make CC0	Non-medical, non-Electron Microscopy images of any kind	"Reference image datasets" - complete, can be associated with other resources, likely to be re-analyzed	Image sets with descriptions and ground truth	Microscopic image sets suitable for image-based profiling
Also non-image data?	Yes	Yes	Yes	Not directly (BioStudies)	Not directly	No	Sometimes
Size limit?	50 GB per collection (soft)	20 GB Figshare, 5TB Figshare+ (soft)	300 GB (soft)	No	1 TB (soft)	No	No
Cost to depositor?	No ("donations encouraged")	Free to $20 GB, $395 to 100 GB, $585/250 GB beyond	$120, + $50/every 10 GB over 50 GB (some funders provide sponsorship)	No	No	No	No
Strictness of metadata requirements	None	Low	Low	Medium	High	Medium	High
