# Supplementary material for: Community-developed checklists for publishing images and image analysis
Source: ArXiv. 2023 Feb 14:arXiv:2302.07005v1. Preprint. [Version 1] (PMC9949169)
Supplement: Supplementary file 1 [file NIHPP2302.07005v1-supplement-1.pdf]

Supplemental materials

Supplemental figure 1. *Alternative layout Checklist for image Publishing*

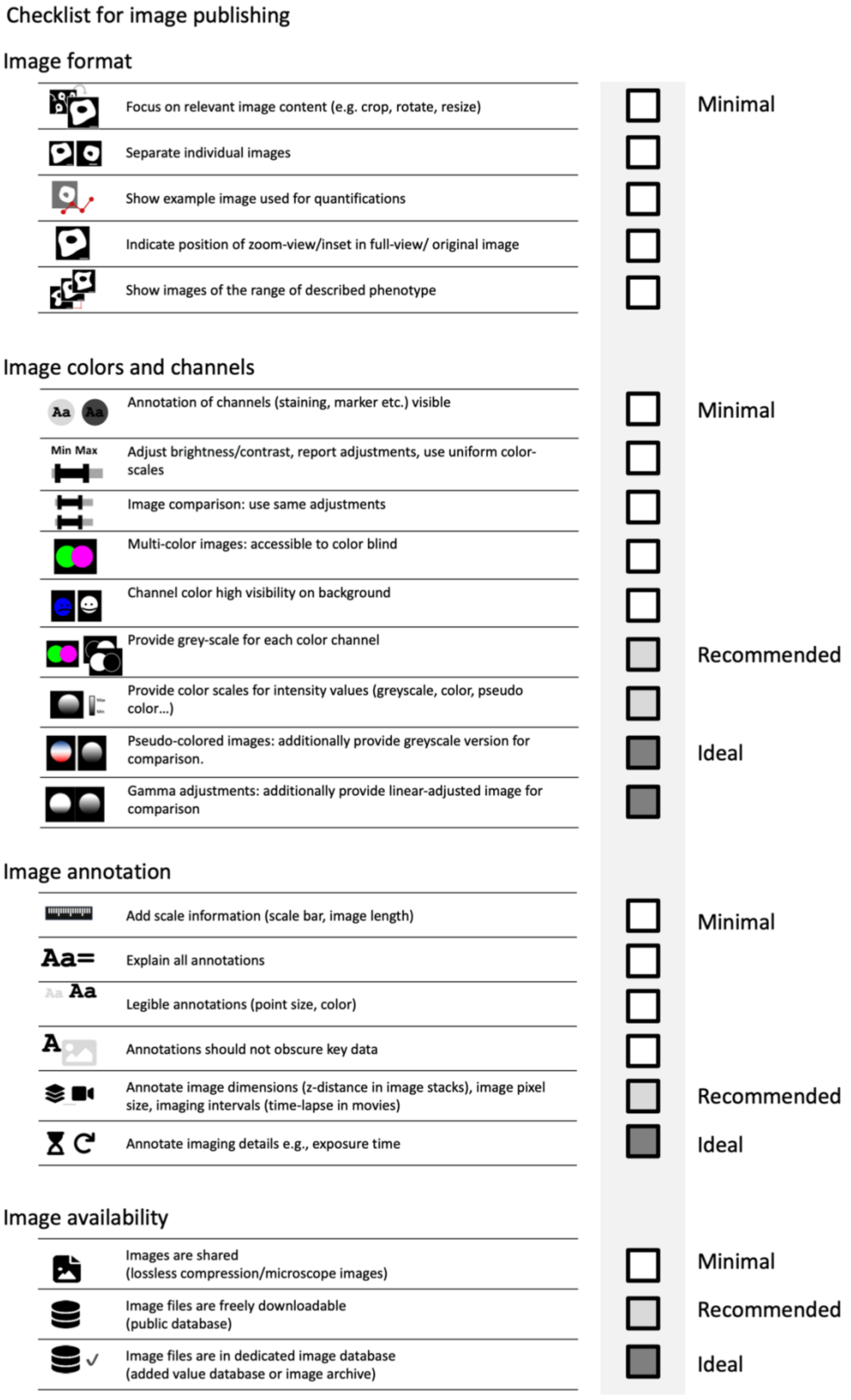

Supplemental figure 2. Alternative layout Checklist for image Analysis Publishing

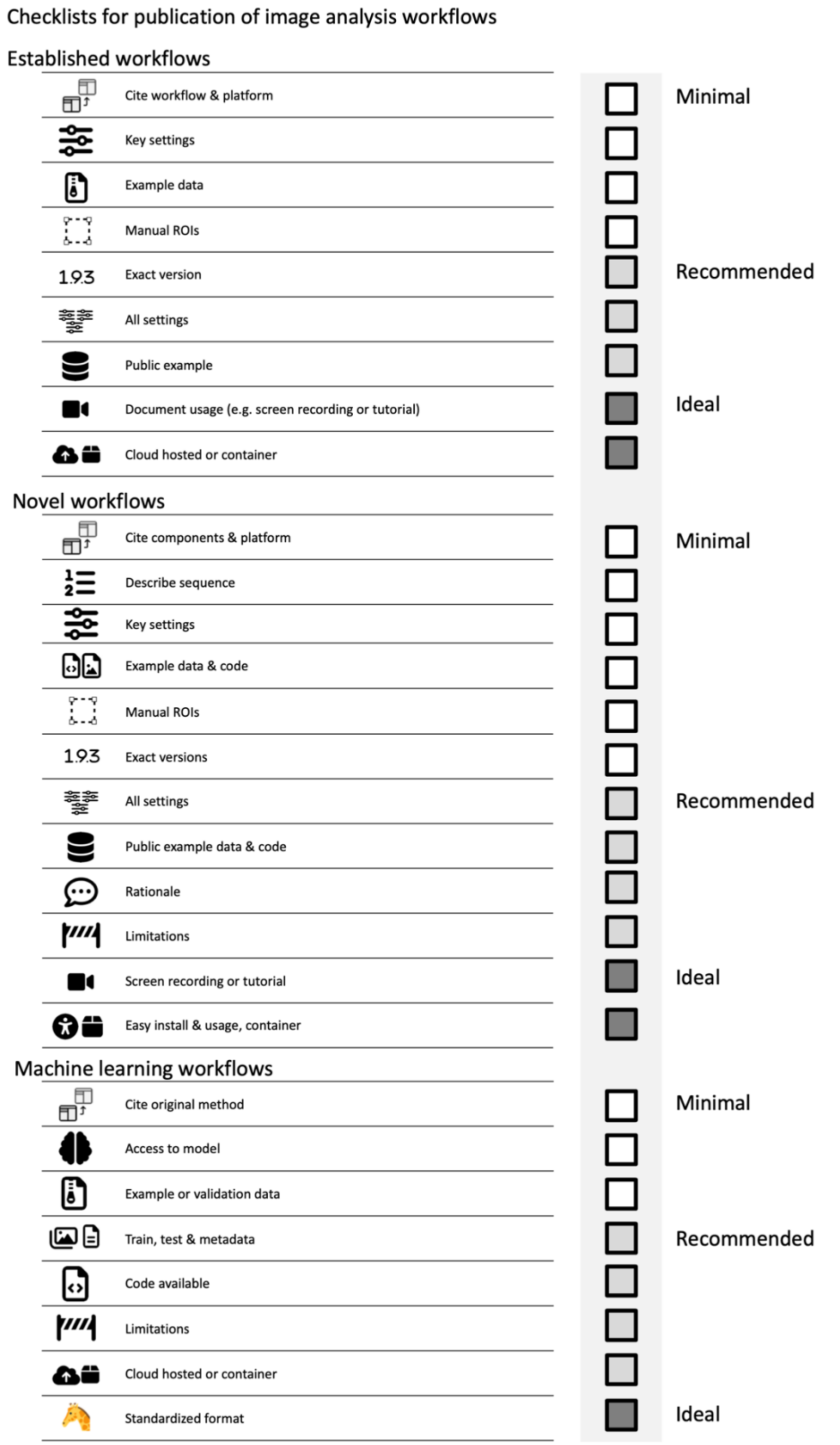

Supplemental figure 3. Overview of current repositories that accept image data. (Cimini, 2023)

Repository	Zenodo	Figshare / Figshare+	Dryad	Bioimage Archive	Image Data Repository	Broad Bioimage Benchmark Collection	The Cell Painting Gallery
URL	https://zenodo.org/	https://figshare.com/	https://datadryad.org/	https://www.ebi.ac.uk/bioimage-archive/	https://idr.openmicroscopy.org/	https://broad.io/BBBC	https://broad.io/cellpaintinggallery
Qualifications	"All the digital artefacts"	Research data outputs	Non-human identifiable data of any kind that authors are willing to make CC0	Non-medical, non-Electron Microscopy images of any kind	"Reference image datasets" - complete, can be associated with other resources, likely to be re-analyzed	Image sets with descriptions and ground truth	Microscopic image sets suitable for image-based profiling
Also non-image data?	Yes	Yes	Yes	Not directly (BioStudies)	Not directly	No	Sometimes
Size limit?	50 GB per collection (soft)	20 GB Figshare, 5TB Figshare+ (soft)	300 GB (soft)	No	1 TB (soft)	No	No
Cost to depositor?	No ("donations encouraged")	Free to $20 GB, $395 to 100 GB, $585/250 GB beyond	$120, + $50/every 10 GB over 50 GB (some funders provide sponsorship)	No	No	No	No
Strictness of metadata requirements	None	Low	Low	Medium	High	Medium	High
